# Body image of university students: a systematic review of the characteristics of interventions

**DOI:** 10.1186/s41155-024-00307-0

**Published:** 2024-06-24

**Authors:** Felipe Machado Huguenin, Vitor Alexandre Rabelo de Almeida, Marcus Vinícius Freitas Rodrigues, Maria Elisa Caputo Ferreira, Fabiane Frota da Rocha Morgado

**Affiliations:** 1 Federal Rural University of Rio de Janeiro, BR-465 Road, Km 7, Seropédica, Rio de Janeiro, 23890-000 Brazil; 2 Federal University of Juiz de Fora, José Lourenço Kelmer Street, São Pedro, Juiz de Fora, Minas Gerais 36036-900 Brazil

**Keywords:** Undergraduate, Training, Program, Body appreciation, Body satisfaction

## Abstract

**Background:**

Body image is the mental representation of the body and can be influenced by cognitive, biological, behavioral, sociocultural, and environmental factors. University students often encounter challenges related to it.

**Objective:**

This systematic review examined interventions aimed at holistically developing a positive body image within this population.

**Methods:**

The PRISMA 2020 guidelines and the PICO method were employed to identify, select, assess, and synthesize studies. The consulted databases included Scopus, Web of Science, and PsycINFO, with inclusion criteria targeting body image interventions for university students aged 18 to 39. Study quality was evaluated using the QATSDD tool.

**Results:**

Twenty-one relevant studies were identified, primarily from the United States, mostly employing quantitative methods, with a focus on female participants. Various intervention strategies were utilized, including cognitive-behavioral approaches, media literacy, and physical/resistance training, with a growing use of technology like mobile applications. The majority of studies reported effective outcomes, such as reduced body dissatisfaction and increased self-esteem following interventions. Nevertheless, literature gaps were identified, such as the scarcity of formative interventions and limited use of qualitative approaches.

**Conclusion:**

While technology in interventions offers promising opportunities, careful assessments and judicious selection of evaluation instruments are fundamental for reliable results. Future research should focus on addressing identified gaps, such as exploring more formative interventions and incorporating qualitative methodologies to provide a more comprehensive understanding of the effectiveness of body image interventions among university students.

## Highlights

• The majority of studies reported effective outcomes, such as reduced body dissatisfaction and increased self-esteem following interventions.

• Nevertheless, literature gaps were identified, such as the scarcity of formative interventions and limited use of qualitative approaches.

• While technology in interventions offers promising opportunities, careful assessments and judicious selection of evaluation instruments are fundamental for reliable results.

## Introduction

Body image is the mental representation of one’s own body (Schilder, 1981). One of the ways to develop, influence, promote, and work on body image is through socio-pedagogical programs, better known as interventions (Carvalho et al., 2014). These refer to initiatives that combine social and educational elements to address specific issues with the aim of promoting personal and social development, aiming not only to provide knowledge or educational skills but also to consider and intervene in the social, emotional, and behavioral dimensions of participants. Additionally, they may count with numerous possible approaches aimed at helping people to establish and flourish a better relationship with themselves, by developing a fully integrated body image (Alleva et al., 2015).

According to Alleva et al. (2015), interventions in body image can be divided into categories. These are (1) cognitive-behavioral, which focuses on the modification of dysfunctional patterns of thoughts, feelings, and behaviors that contribute to the formation of negative body image; (2) media literacy, which aims to develop the individual’s ability to critically analyze images and messages conveyed by the media; (3) psychoeducation, which has the objective of teaching people about body image, its causes, and implications; (4) physical training, which englobes aerobic and anaerobic activities intending to enhance fitness; and (5) promotion of self-esteem, by highlighting that low self-esteem is strongly associated with the development of negative body image.

It is important to highlight other types of approaches do not perfectly fit into one of the aforementioned categories, such as yoga, in which three elements are fundamental: controlled breathing, mediation techniques, and physical postures or poses (e.g., Ariel-Donges et al., 2019); creative psychotherapy, a creative process in which body movements are used as a facilitator to explore and integrate emotional, cognitive, physical, social, and spiritual facets of individuals (e.g., Russell-Mayhew et al., 2012); or mindfulness, which is composed of meditation practices that emphasize observations of thoughts and feelings free of judgment, encouraging the achievement of a receptive, peaceful, and present state of mind (e.g., Adams et al., 2013; Díaz-Ferrer et al., 2017; Toole & Craighead, 2016).

Accordingly, it has been demonstrated it is important to consider these body image interventions both, to prevent alterations in this construct, primary intervention, and to mitigate causes and risk factors, and secondary intervention (Williamson et al., 2018). Guest et al. (2019) carried out a systematic review of the efficacy of interventions aimed at promoting positive body image in adults. The study had the objective of examining the interventions used to enhance the perception of body image and evaluate their efficacy. The results suggest that a variety of interventions may have a positive impact on the promotion of a positive boy image in adults. However, the heterogeneity of included studies makes it difficult to directly compare them. Additionally, the authors stress the necessity of diverse approaches and high-quality investigations to deeply understand the effects of these interventions (Guest et al., 2019).

Similarly, Yager et al. (2013) conducted a systematic review aimed at evaluating the efficacy of body image programs delivered to adolescents at their schools. The authors analyzed many body image programs focused on preventing body dissatisfaction, eating disorders, and appearance-related social pressures of adolescents in secondary schools. Results indicate that prevention programs may have a positive influence on the attitudes of adolescents toward their bodies, but the efficacy varies considerably among the evaluated programs. The authors highlight the importance of adapting programs to the school context to achieve significant results in the promotion of positive body image among adolescent students (Yager et al., 2013).

A more recent study conducted by Mahon and Seekis (2022) systematically reviewed digital interventions aimed at enhancing the body image of adolescent girls and young women. The research aimed to comprehensively analyze digital interventions that aim to address issues related to body image in this population. The results indicated the growing relevance of digital interventions as potential tools to influence positive body image. However, the authors noted the need for more research to assess the long-term effectiveness of interventions and to better understand the factors that contribute to the success of approaches in this context.

In Brazil, so far, studies have confirmed the relevance of risk factors identified in other cultures, such as media influence, body dissatisfaction, internalization of beauty standards, depression, and diet, but few studies have evaluated evidence-based programs (Hudson et al., 2021). However, promoting socio-pedagogical programs for diverse populations is as important as investigating and analyzing the multiple facets of body image, along with its associations (Carvalho et al., 2014).

A population deserving attention is that of young university students. Due to its transitional nature, marking the transition from high school to college, a series of lifestyle transformations in these young individuals occur significantly. Such changes take place in conjunction with the pressures and challenges inherent in this phase of the life cycle, along with the potential connection between concerns related to body weight and physical conformation (Howard et al., 2020). The body image of these students is closely linked to the beauty standard, where a slim body is seen as beautiful for women, and a mesomorphic body is desired by men. In this sense, the literature indicates that young university students may exhibit signs and characteristics of negative body image (Chin et al., 2020).

Although some reviews have provided valuable contributions by identifying effective interventions aimed at promoting positive body image in various contexts and populations, they have not been able to provide a comprehensive understanding of body image interventions in the university student population. Consequently, the ability to substantively address the queries of teachers, educational institutions, policymakers, and government bodies regarding effective approaches in this scenario remains a challenge.

In this regard, the identified gap in the literature regarding interventions aimed at promoting positive body image among college students lies in the lack of comprehensive studies that extensively investigate the effectiveness and adaptation of specific programs for this demographic group, highlighting a need for research to inform practices and policies aimed at these young adults. Thus, systematically understanding studies investigating the body image of university students to deepen knowledge in the field and enable the promotion of more positive and effective socio-pedagogical programs in education is crucial. This article aims to fill this identified gap in the literature through a systematic review. Therefore, we intend to analyze the characteristics of scientific production on interventions focusing on the body image of university students.

## Method

### Research strategy

This systematic review is registered in the International Prospective Register of Systematic Reviews (PROSPERO)/National Institute for Health Research, under the number CRD42022384998 and follows the Preferred Reporting Items for Systematic Reviews and Meta-Analyses (PRISMA) 2020 guidelines, statement, designed to transparently report why the review was conducted, what the authors did, and what they found, i.e., methods for identifying, selecting, evaluating, and synthesizing studies (Page et al., 2021).

Given the multidisciplinary nature of the body image field, the following databases were searched: Scopus, Web of Science, and PsycINFO accessed through the Federated Academic Community (CAFe) on the portal of journals of the Coordination for the Improvement of Higher Education Personnel of the Ministry of Education of Brazil (CAPES/MEC). There was no time restriction for the literature search, which was completed on April 2024. The development of the central research question allows the delimitation of necessary information, maximizing evidence retrieval in databases and avoiding unnecessary searches. Therefore, the PICO strategy was chosen, an acronym for P: population, I: intervention, C: comparison/control, and O: outcome (Santos et al., 2007).

In the three databases, studies were identified using the following terms in the title, abstract, and keywords: P) college OR university OR undergraduate OR “pre-service teachers”; I) teaching OR learning OR instruction OR training OR “educational development”; C) intervention OR program OR project; O) “body image” OR “self-image” OR “body identity” OR “body representation” OR “body schema” OR “body appreciation” OR “body *satisfaction”. Initially, the article selection process in the databases was conducted by two researchers based on the examination of titles, abstracts, and keywords. In cases of disagreement, researchers engaged in dialogue, and in the persistence of discordance, a third researcher was consulted.

### Data extraction

The inclusion criteria used in this systematic review were as follows: (1) selection of articles that aimed at implementing a body image intervention, (2) studies targeting the university student population, (3) articles involving an age range between 18 and 39 years, due to the relevance of the transitions period to adulthood and the consequent significant changes in the perception of body image and identity formation within this age range. Additionally, middle adulthood begins at the age of 40 (Carvalho et al., 2014), which justifies focusing studies on the age range between 18 and 39 years.

The exclusion criteria were carefully delineated to filter out studies that were not aligned with the specific objectives of the systematic review. The following studies were excluded: (1) articles targeting populations with specific clinical conditions; (2) studies applying an intervention to an adult population without specifying that they were university students; (3) studies focusing on eating disorders or mood disorders, regardless of body image measurements; and (4) articles in languages other than English, Spanish, and Portuguese, with the aim of ensuring accessibility and comprehension of the texts by the researchers.

Duplicate articles found in the analyzed databases were also excluded, as well as book chapters, dissertations, theses, review articles, and articles not directly related to intervention, body image, and university students. Furthermore, this systematic review has not included additional sources such as grey literature and conference proceedings. For each selected study, the following data were extracted: authors, year of publication, country in which the study was carried out, intervention name, population, intervention resource, intervention approach, number of sessions, follow-up, experimental/control group, instruments, objective, and conclusion.

### Article quality

To assess the quality of the studies included in this systematic review, the Quality Assessment Tool for Reviewing Studies with Diverse Designs (QATSDD), developed by Sirriyeh et al. (2012), was employed. The data used for the assessment were directly extracted from the articles included in this study. The choice of this quality assessment tool was grounded in its strengths and its ability to provide a comprehensive and standardized assessment of the methodological quality of studies, both qualitative and quantitative. The QATSDD is recognized for its capacity to evaluate studies with different research designs, which is especially relevant in systematic reviews that may encompass a variety of studies. Moreover, the tool demonstrates good reliability and validity, offering a consistent and rigorous approach to evaluating the quality of included studies. However, it is important to acknowledge that the QATSDD also has limitations; for instance, the interpretation of tool items may vary among assessors, and it may not capture all the nuances of methodological quality in certain studies, particularly in complex or emerging research areas (Sirriyeh et al., 2012).

Each study received a score on a scale of 0 to 3 points for each of the 16 items. A minimum score of 0 indicates that the authors did not provide any information related to the item in question. A 1-point score indicates minimal mention of the information, while a 2-point score indicates that the information was partially provided in the study. The maximum score of 3 points was assigned when the authors presented the information in a complete and detailed manner. It is worth noting that this process was conducted independently by the same two researchers responsible for the article selection, and cases of disagreement were resolved through dialogue between them.

The quality of each study was calculated based on the percentage of the maximum score achieved. The maximum possible score is 48 points for mixed-method studies; for studies that are purely quantitative or purely qualitative, the maximum score is 42 points. Studies that scored above 50% were classified as having good or high quality. Conversely, studies that did not reach this percentage were considered of lower-than-expected quality, according to the criteria established by Sirriyeh et al. (2012), and were consequently excluded from this systematic review.

The quality of the studies plays a fundamental role in the reliability and interpretation of the results of this systematic review. An assessment of the methodological quality of the included studies was essential to ensure more accurate conclusions. Lower scores in the quality assessment may indicate significant flaws in research procedures, such as lack of variable control, inadequate sampling, or selection bias. Such deficiencies can compromise the robustness of the results, leading to erroneous or imprecise conclusions regarding the effectiveness of interventions on the body image of university students. Furthermore, the lack of methodological detail can hinder the replication of studies and the understanding of the underlying mechanisms of the observed effects. Therefore, when interpreting the results of this review, it is crucial to consider the quality of the studies as a critical aspect that can directly influence the conclusions and recommendations derived from this analysis.

## Results

**Fig. 1:**
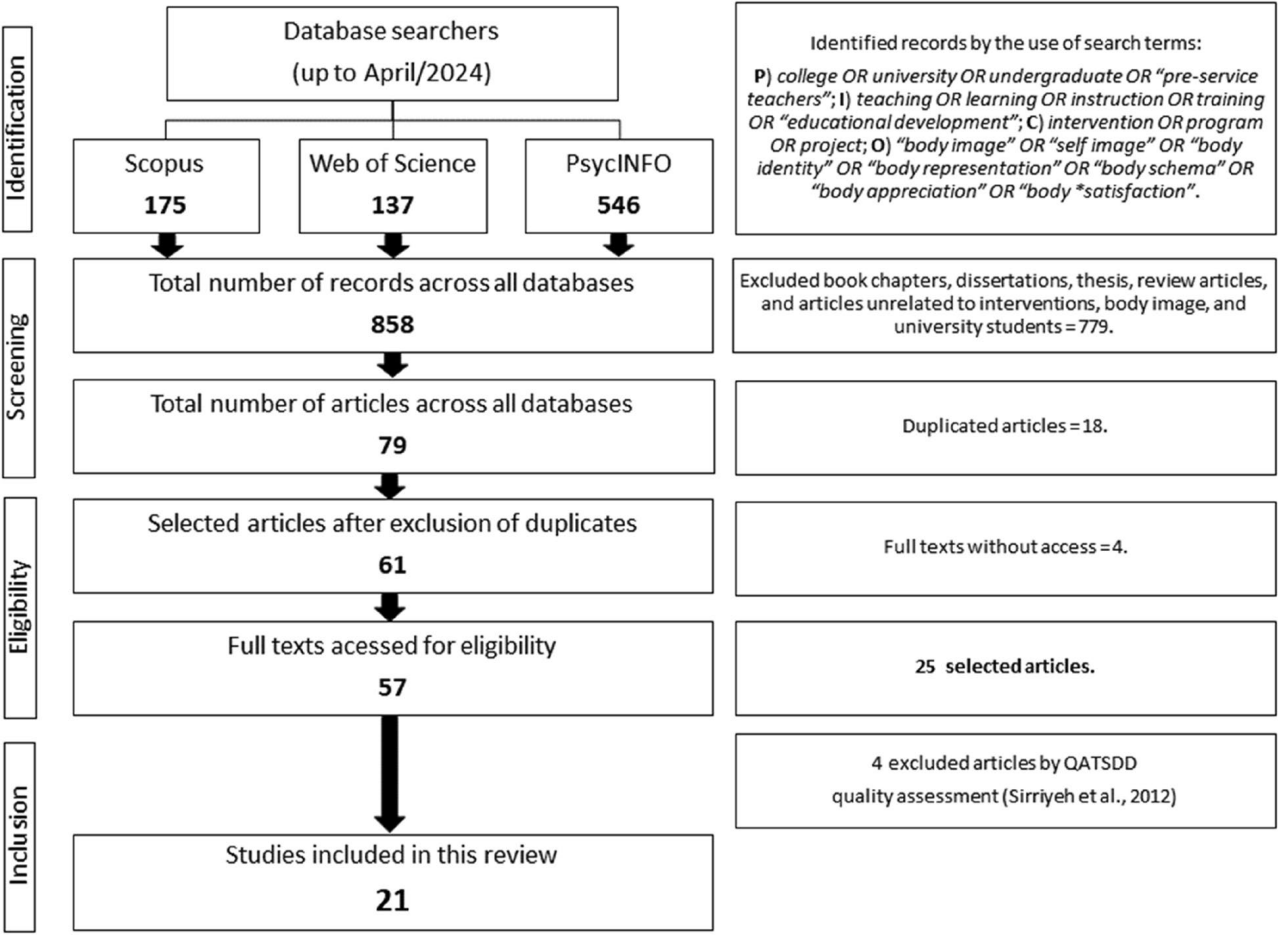
Flowchart of the systematic process of identification and selection of studies

Figure 1 outlines the procedures adopted in the selection of studies for analysis in this review. Initially, a comprehensive survey was conducted, resulting in the identification of 858 relevant records. Subsequently, exclusion criteria were rigorously applied, leading to a final sample of 21 publications that underwent detailed analysis.

Table 1 provides a detailed description of the studies found in this systematic review. The column “#” lists the sequential number of the studies. “Authors/year of publication/country” identifies the authors, the year of publication, and the country of origin of the participants. “Name of intervention” describes the name of the intervention applied in the study. “Population” indicates the target population, such as the age and gender of the participants. “Intervention resource” specifies the resources used in the intervention. “Intervention approach” details the intervention approach, such as cognitive-behavioral or other methods. “Sessions” provides the number of sessions conducted during the intervention. “Follow-up” describes the post-intervention follow-up period to evaluate the results. “Groups Exp. X Control” indicates the existence of a comparison between the experimental group and the control group. “Instruments” lists the measurement instruments used, such as psychometric scales. “Objectives” defines the objectives of each study. “Conclusion” summarizes the main conclusions found by the researchers. Finally, “QATSDD” presents the quality score of the study according to the “Quality Assessment Tool for Studies with Diverse Designs.”

**Table 1:** Studies on interventions in the body image of university students

No	Authors/year of publication/country	Name of intervention	Population	Intervention resource	Intervention approach	Sessions	Follow-up	Groups Exp. X control	Instruments	Objectives	Conclusion	QATSDD
1	Kosma et al. (2024) USA	Physical theater	8 participants	Classes *Integrates an academic semester*	Physical activity	1 academic semester *(2* × */week; 90 min each)*	No follow-up	No control group	Semi-structured interviews	Examine the effects of a semester-long physical theater class on body schema (body posture, awareness, confidence, expression)	Students highlighted the importance of physical theater to improve body posture, confidence, and emotional expression, emphasizing the need for integrated and holistic movement programs to enhance the body schema	24 57%
2	Bolter et al. (2023) USA	Brief weight bias, pedagogical intervention	Women, men, transgender men, transgender women, gender non-conforming subjects (*n* = 81)	Classes *Integrates an academic semester*	It did not indicate	3 modules, which could be taught between weeks 4 and 9 of the academic semester	No follow-up	Exp. = 48 Cont. = 33	AFAQ FPS BAS Semi-structured interviews	To evaluate the impact of a pedagogical intervention on weight stigma outcomes among first-year students	It highlights the importance of pedagogical interventions, showing that although the quantitative results were not significant, the qualitative ones suggest that brief interventions integrated into existing courses have the potential to engage students, indicating the need for longer and more uniform interventions, focusing on intersectionality and less stigmatizing measures of body composition	36 75%
3	Ong and Sündermann (2022) Singapore	Intellect *Mental health app*	Women and men (*nW* = 213; *nM* = 97)	Mobile app training	Cognitive-behavioral (cognitive dissonance) Psychoeducation Media literacy	9 days *(*< *5 min/day)*	4 weeks	Exp. = 149 Cont. = 161	BIQ BAS-2 SATAQ-4 SCS;	To examine the effectiveness of a self-guided mobile health (mHealth) app in improving body image and self-compassion in a sample of university students	It presents preliminary evidence that a self-guided mHealth app may contribute to the improvement of body image concerns and self-compassion in young adult university students	33 79%
4	Cerea et al. (2021) Italy	GGBI *Cognitive training of a mobile app*	Women (*nM* = 50)	Mobile app Cognitive training	Cognitive-behavioral	16 days (3 min/day)	32 days T0–T1 = 16 days T1–T2 = 16 days	Exp. = 25 Cont. = 25	EDI-3 DASS-21 QDC	To evaluate the effectiveness of a mobile application based on cognitive-behavioral principles in reducing body dissatisfaction and symptoms of body dysmorphic disorder/eating disorders in female university students	Training can lead to reductions in some forms of body dissatisfaction, including symptoms of body dysmorphic disorder, in female university students at high risk of developing body image disorders	33 79%
5	Glashouwer et al. (2020) Netherlands	AAT *Personalized avoidance training*	Women (*nM* = 104)	Computer *Task training*	Cognitive-behavioral	4 sessions	1 week	Exp. = 34 Cont. placebo = 35 Cont. no training = 35	VAS BISS DT EDE-Q	To test whether training in approach and avoidance tendencies related to thinness is effective in improving body satisfaction through the use of personalized training with individuals’ body images	It did not provide evidence that approach-avoidance training is effective in improving body image	21 50%
6	Dondzilo et al. (2020) Australia	Modification of attention bias based on touchscreen	Women (*nW* = 110)	Computer *Task training*	Cognitive-behavioral	1 sess	No follow-up	No control group	DASS-21 BSRI	To determine the direct effect of attention bias of bodies with internalized thinness ideal on the state of depressive rumination and evaluate the efficacy of the attention bias using a touchscreen device	Current discoveries suggest that the modification of attention bias based on the touchscreen is effective in modifying patterns of attention bias and state of depressive rumination	22 52%
7	Wilver et al. (2020) USA	Safety behavior fading	Women (*nW* = 84)	Meetings *Training through instructions*	It did not indicate	2 s	1 week	Exp. = 41 Cont. = 43	EDI-3 BDD-YBOCS-SR SPIN BAAS IQ CES-D ABC	To explore the experimental effects of involvement reduction in behaviors related to appearance in appearance concerns and related symptoms	It provides robust evidence for the importance of safety behaviors related to appearance in the maintenance of body dysmorphic disorder and other body image-related disturbances	33 79%
8	Rato and Alves (2020) Portugal	SM *Somatic movement*	Women and man (*nW* = 9; *nM* = 1)	Classes *It integrates the dance curriculum plan*	Psychoeducation	20 sessions *(1 semester)*	No follow-up	No control group	Semi-structured interviews in depth; interviews of explanation; participants’ diaries and group discussions	To study the body image of dance students, through the development and application of a Somatic Movement program in a group of undergraduate dance students	The conscious utilization combined with multiple methods, such as in-depth semi-structured interviews; explanation interviews, group discussions, participants’ diaries, and field notes of the researcher brought significant advantages during the intervention	24 57%
9	Aboody et al. (2020) Israel	Mobile app for body image resilience	Women (*nW* = 91)	Mobile app *Training*	Cognitive-behavioral	14 days *(4 min/day) (up to level 54)* + a task of resilience on Instagram	1 month	Randomization 1:1	BAS-2 VAS PASTAS DASS-21 BIDQ SISE	To evaluate the effects of a cognitive-behavioral-based mobile app, projected to enhance the resilience to body image triggers and to reduce the symptoms of body image perturbances	The results highlight the potential utility of brief portable interventions of low intensity in reducing symptoms of body image perturbances and enhancing resilience to messages of thin bodies often propagated on social media	28 67%
10	Glashouwer et al. (2019) Netherlands	CE *The procedure of evaluative conditioning*	Women (*nW* = 129)	Computer *Task training*	It did not indicate	2 sessions *(30 min each)*	No follow-up	Exp. = 67 Cont. = 62	VAS BISS EDE-Q SSES RSES	To investigate if a procedure of evaluative conditioning based on computer use, utilizing social positive feedback is effective for improving body satisfaction	It suggests that the procedure of evaluative conditioning in its current format is not ready to be used as an intervention for improving body satisfaction	29 69%
11	Ariel-Donges et al. (2019) USA	Yoga	Women (*nW* = 75)	Meetings *Yoga*	Yoga	12 weeks *(2* × */week; 60 min each)*	No follow-up	Exp. = 37 Cont. = 38	MBSRQ-AS BDI-2 EAT-26 FMI	To evaluate the efficacy of Yoga as an innovative treatment for body dissatisfaction in young university women	It suggests that Yoga could help young university women to develop a healthier relationship with their bodies	30 71%
12	Lee et al. (2019) Taiwan	Beauty in the middle of movement: my healthy body image	Women (*nW* = 57)	Meeting *Creative movement*	Creative psychotherapy	8 sessions *(1* × */week; 90 min each)*	3 months	Exp. = 16 Cont. E. F. = 17 Cont. = 24	CES-D RSES MBSRQ-AS FFMQ	To reduce body dissatisfaction in female university students	It shows that after the program, scores of body image, mindfulness, and self-esteem improved in the experimental group in comparison to the other comparison and control groups. Scores remained elevated for 3 months after the intervention	26 62%
13	Díaz-Ferrer et al. (2017) Spain	Pure and guided exposure to the mirror	Women (*nW* = 35)	Meeting *Mirror exposure training*	Mindfulness	6 sessions *(2* × */week; 45 min each)*	No follow-up	Exp. pure = 17 Exp. guided = 18	VAS BIATQ BIAQ	To examine the psychophysiological alterations resulting from two treatments of mirror exposure that showed efficacy in reducing body dissatisfaction	It suggests that both approaches are effective as interventions to improve body image disturbances. However, the psychophysiological alterations observed during the sessions suggest that each technique may operate through different processes	28 67%
14	Toole and Craighead (2016) USA	Self-compassion meditation	Women (*nW* = 80)	Meetings *Meditation*	Mindfulness	1 week *(20 min/day)*	No follow-up	Exp. = 40 Cont. = 40	BAS-2 SCS RSES OBCS CSW BSQ	To evaluate an online-based version of self-compassion training	It suggests that brief exposure to the basic principles of self-compassion has the potential to improve aspects of self-compassion and discomfort with body image	23 55%
15	Khazir et al. (2015) Iran	Media literacy	Women (*nW* = 140)	Meetings *Media literacy training*	Media literacy	4 weeks 4 sessions *(40 to 60 min)*	4 weeks	Exp. = 70 Cont. = 70	RSES *Acceptance of Cosmetic Surgery* *Scale* BSS BICI	To examine the favorable attitude of a group of female university students about elective cosmetic surgery, body dysmorphic disorder, self-esteem, and body dissatisfaction after an intervention of media literacy training	It emphasizes the importance of media literacy intervention in reducing women’s favorable attitudes toward elective plastic surgery, body dysmorphic disorder, and body dissatisfaction, as well as enhancing self-esteem	33 79%
16	Becker et al. (2013) Estados Unidos	PL-DBI *Peer-led cognitive dissonance-based intervention*	Women (*nW* = 92)	Meetings *Sessions of training*	Cognitive-behavioral *(cognitive dissonance)*	2 sessions *(2 h each)*	8 months	No control group	EDE-Q OBCS IBSS-R BPS PANAS	To investigate whether an evidence-based body image improvement program, targeting the internalization of a thin ideal among college women also reduces self-objectification	It provides preliminary support for the use of dissonance interventions in reducing self-objectification and body control beliefs	26 62%
17	Adams et al. (2013) USA	Brief mindfulness	Women (*nW* = 65)	Meetings *Meditation*	Mindfulness	1 session	No follow-up	Purse + silence = 16 BS + silence = 15 Purse + mindfulness = 15 BS + mindfulness = 18	VAS EAT-26 FFMQ BSQ PANAS SSQ SOC BULIT-R MAEDS TMS QSU	To examine whether mindfulness can be a useful technique for minimizing the influence of body dissatisfaction on negative affect, smoking cravings, and smoking behavior	It provides preliminary support for the use of mindfulness-based treatments for female smokers in addressing body dissatisfaction	31 74%
18	Russell-Mayhew et al. (2012) Canada	Workshop of interactive training	Women and men (*nW* = 10; *nM* = 6)	Workshop	Interactive training	1 session *(3 h)*	3 months	No control group	SATAQ EAT BSS	To examine body image satisfaction and eating/weight-related behaviors before and after a professional workshop with future physical education teachers	Providing professional development for pre-service teachers can promote a more positive approach to body image, weight bias, and weight/eating-related concerns in schools	24 50%
19	Moore et al. (2011) USA	Program of 12-week resistance training	Women and men (*nW* = 37; *nM* = 83)	Classes *University resistance training course (semester)*	Physical training	12 weeks *(2* × */week)*	No follow-up	No control group	RSES PSPP PSAQ	To evaluate self-esteem using the hierarchical structure of the Exercise and Self-Esteem Model	It indicates significant improvements in self-perception constructs across all levels of the Exercise and Self-Esteem Model	21 50%
20	Yager and O’Dea (2010) Australia	Controlled intervention to promote healthy body image	Women and men (*nW* = 110; *nM* = 60)	Classes *Regular didactic program*	Cognitive-behavioral *(cognitive dissonance)* Media literacy	12 weeks (per intervention)	6 months *(T3)*	T1 and T2/T3: Exp. 1 = 52/20 Exp. 2 = 49/42 Cont. = 69/29	EDI EDE-Q GSW DEBQ BAR	To examine the impact of two interventions on body image, risk of eating disorders, and excessive exercise in 170 pre-service physical education and health teachers	It is feasible to promote body image, reduce body dissatisfaction, and decrease excessive exercise among pre-service physical education and health teachers through a health education curriculum	27 64%
21	Henry et al. (2006) USA	Aerobic and circuit training	Women (*nW* = 72)	Meetings *Physical exercise*	Physical training	12 weeks (3 × /weeks; 50 min each)	No follow-up	Aerobic training = 23 Circuit training = 28 Cont. = 21	BSIQ;	To determine the effect of aerobic and interval circuit training on the physical fitness and body image of women	It was concluded that an interval program combining aerobic, anaerobic, and strength training is more beneficial for improving body image than solely engaging in aerobic exercises or no exercise at all	29 69%

*ABC* Appearance Behavior Checklist, *AFAQ* Anti-Fat Attitude Questionnaire, *BAAS* Beliefs About Appearance Scale, *BAR* body appearance rating, *BAS* Body Appreciation Scale, *BAS-2* Body Appreciation Scale-2, *BDD-YBOCS-SR* Body Dysmorphic Disorder-Yale-Brown Obsessive–Compulsive Scale, *BDI-2* Body Dissatisfaction Inventory-2, *BIAQ* Body Image Avoidance Questionnaire, *BIATQ* Body Image-Acceptance and Action Questionnaire, *BICI* Body Image Coping Strategies Inventory, *BIDQ* Body Image Disturbance Questionnaire, *BIQ* Body Image Questionnaire, *BISS* Body Image States Scale, *BPS* Body Parts Satisfaction Scale, *BSIQ* Body Shape Interrogation Questionnaire, *BSQ* Body Shape Questionnaire, *BSRI* Brief State Rumination Inventory, *BSS* Body Satisfaction Scale, *BULIT-R* Bulimia Test-Revised, *CES-D* Center for Epidemiologic Studies Depression Scale, *CSW* cultural standards for women, *DASS-21* Depression Anxiety Stress Scale-21, *DEBQ* Dutch Eating Behaviors Questionnaire, *DT* drive for thinness, *EAT* Eating Attitudes Test, *EAT-26* Eating Attitudes Test-26, *EDE-Q* Eating Disorder Examination Questionnaire, *EDI* Eating Disorder Inventory, *EDI-3* Eating Disorder Inventory-3, *FPS* Fat Phobia Scale, *FFMQ* Five Facet Mindfulness Questionnaire, *FMI* fat mass index, *GSW* global self-worth, *IBSS-R* Interpersonal Body Comparison Scale-Revised, *IQ* interpretation questionnaire, *MAEDS* multidimensional assessment of eating Disorders Symptoms, *MBSRQ-AS* Multidimensional Body-Self Relations Questionnaire, *OBCS* Objectified Body Consciousness Scale, *PANAS* Positive and Negative Affect Schedule, *PASTAS* Physical Appearance State and Trait Anxiety Scale, *PSAQ* Physical Self-Appreciation Questionnaire, *PSPP* Physical Self-Perception Profile, *QDC* Questionnaire on Dysmorphic Concerns, *QSU* Questionnaire of Smoking Urges, *RSES* Rosenberg Self-esteem Scale, *SATAQ* Sociocultural Attitudes Toward Appearance Questionnaire, *SATAQ-4* Sociocultural Attitudes Toward Appearance Questionnaire-4, *SCS* sociocultural standards, *SISE* Single-Item Self-Esteem Scale, *SOC* sense of coherence, *SPIN* Social Phobia Inventory, *SSES* Self-Esteem Scale, *SSQ* Self-Stat Body Image Questionnaire, *TMS* thinness motivation scale, *VAS* visual analog scale

One of the outcomes was the exclusion of four articles primarily because their scores according to the QATSDD indicated significant methodological deficiencies. These deficiencies included inadequate control of variables, poor sampling techniques, and potential biases in participant selection, all of which could lead to premature conclusions about the efficacy of body image interventions. Additionally, these articles lacked sufficient methodological detail, complicating the replication and understanding of the mechanisms behind the observed effects.

It is noteworthy that more recent interventions utilize digital technology as a resource in an attempt to broaden the reach to a broader and more diverse population, and that the inclusion of interventions during the academic semester in undergraduate courses has been a commonly used approach.

Conversely, Table 2 synthesizes information regarding the participants’ country of origin, study population, research method employed, intervention approach, and instruments applied. To provide detailed insights into the most recurring information, absolute and relative frequencies were calculated.

**Table 2:** Characterization of studies

**Characteristics: specificities (article number is relative to **Table 1**)**	**Quantity**	**%**
The origin country of the participants		
USA (1, 2, 7, 11, 14, 16, 17, 19, 21)	9	42.9%
Australia (6, 20)	2	9.5%
Netherlands (5, 10)	2	9.5%
Other (3, 4, 8, 9, 12, 13, 15, 18)	8	38.1%
Population		
Women (4, 5, 6, 7, 9, 10, 11, 12, 13, 14, 15, 16, 17, 21)	14	66.7%
Women and men (1, 3, 8, 18, 19, 20)	6	28.6%
Self-declared gender identity (2)	1	4.8%
Study design		
Quantitative (3, 4, 5, 6, 7, 9, 10, 11, 12, 13, 14, 15, 16, 17, 19, 20, 21)	17	81.0%
Qualitative (1, 8)	2	9.5%
Mixed methods (2, 18)	2	9.5%
Intervention approach		
Cognitive-behavioral (3, 4, 5, 6, 9, 16, 20)	7	33.3%
Media literacy (3, 15, 20)	3	14.3%
Mindfulness (13, 14, 17)	3	14.3%
Psychoeducation (3, 8)	2	9.5%
Physical training (19, 21)	2	9.5%
Physical activity (1)	1	4.8%
Yoga (11)	1	4.8%
Creative psychotherapy (12)	1	4.8%
Interactive training (18)	1	4.8%
Most used instruments		
RSES: Rosenberg Self-Esteem Scale (10, 12, 14, 15, 19)	5	26.3%
EDE-Q: Eating Disorder Examination Questionnaire (5, 10, 16, 20)	4	21.1%
EDI: Eating Disorder Inventory (4, 7, 20)	3	15.8%
BAS-2: Body Appreciation Scale-2 (3, 9, 14)	3	15.8%
DASS-21: Depression Anxiety Stress Scale-21 (4, 6, 9)	3	15.8%
BSS: Body Satisfaction Scale (15, 18)	2	10.5%
MBSRQ: Multidimensional Body-Self Relations Questionnaire (11, 12)	2	10.5%
EAT-26: Eating Attitudes Test-26 (11, 17)	2	10.5%
SATAQ: Sociocultural Attitudes Toward Appearance Questionnaire (3, 18)	2	10.5%

In relation to the country of origin of participants, it is noteworthy that a significant number of studies were conducted in the United States, contributing to nine studies. Regarding the study populations, 14 studies exclusively focused on female participants, while in the remaining studies, participants included both females and males. It is important to highlight that in only one study, the number of male participants exceeded that of females (e.g., Moore et al., 2011), and only one study utilized self-declared gender identity among the participants (e.g., Bolter et al., 2023).

Trends in intervention approaches to improve body image show variations over time and across different geographic regions. In the United States, a diversity of methods is observed, with a particular emphasis on cognitive-behavioral approaches, mindfulness-based interventions, and physical activities. Notably, the cognitive dissonance technique is frequently employed, reflecting a focus on modifying dysfunctional thoughts and behaviors. In Europe, countries such as the Netherlands and Italy also predominantly adopt cognitive-behavioral approaches, suggesting an acceptance of this methodology in the region. In contrast, regions like Asia and the Middle East show a preference for interventions such as creative psychotherapy and media literacy, respectively, indicating a cultural adaptation of strategies. Notably, recent interventions in various regions are incorporating digital technologies, expanding the reach and accessibility of interventions. Additionally, the increasing inclusion of mindfulness and yoga practices reflects a global trend toward integrative approaches.

As for the research methods employed, 17 studies adopted a quantitative approach, 1 study opted for a qualitative form, and another research used a mixed qualitative-quantitative method. Concerning the intervention approaches, the cognitive-behavioral approach emerges notably, observed in seven trials. It is relevant to note that the use of the cognitive dissonance technique was noted in three distinct studies, emphasizing its application in the context of the addressed interventions (e.g., Becker et al., 2013; Ong & Sündermann, 2022; Yager & O’Dea, 2010).

Additionally, it was found that the most frequently used psychometric measure in the interventions of the analyzed articles was the Rosenberg Self-Esteem Scale (RSES), which can be found in various studies in the field of body image (e.g., Kerner et al., 2018; Tiggeman & Zinoviev, 2019).

**Fig. 2:**
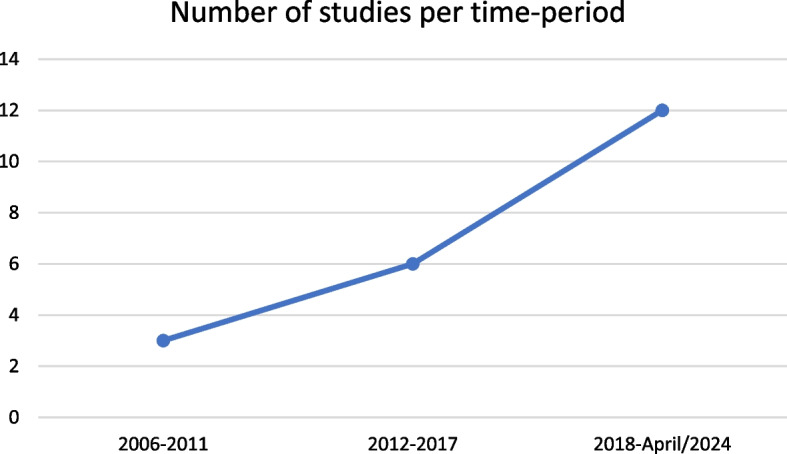
Chronological evolution of studies included in this review

Most of the articles were published between 2018 and 2022, indicating a considerable increase in interest in exploring this theme in recent years (Fig. 2). These findings contribute to the understanding of the current landscape of studies on interventions focusing on the body image of university students, providing a broad overview of the predominant methodological and thematic characteristics.

## Discussion

The primary objective of this systematic review was to analyze the characteristics of scientific production on interventions focusing on the body image of university students. We examined each intervention, considering relevant aspects such as the country of origin of the population, the gender of participants, methods employed in each study, and assessment instruments used in the interventions. However, we will focus our attention on four topics that we consider most relevant, with the aim of specifically analyzing them: (1) intervention approach; (2) sessions, follow-up, and experimental and control groups; (3) objectives and conclusion; and (4) intervention resources. These insights will contribute to a more in-depth understanding of contemporary scientific production.

Regarding the countries of origin of study participants, the scarcity of publications from Brazil stands out. On the other hand, the United States stands out for the considerable number of studies conducted, contributing to almost 42.9% of the total. Australia and the Netherlands also deserve mention, with each country contributing to two studies. These data align with the findings of Andersen and Swami (2021), who analytically indicated that most articles on body image come from Anglo-Saxon countries. Although these findings highlighted by the authors are from articles published in the *Body Image* journal, they provide a broad perspective as the journal is a global reference in the field.

While the representation of other countries is relatively smaller compared to the United States, it demonstrates interest from various nations in researching interventions focusing on the body image of university students. The findings suggest that interventions are not restricted to a specific geographical region, indicating that this theme is relevant in different cultural contexts. This regional diversity in methodologies underscores the importance of considering cultural and social contexts in the selection of interventions, promoting more effective and sensitive strategies to the specific needs of the populations.

The analysis of studies revealed important patterns in terms of population composition. Most studies focused exclusively on women, reflecting a prominent emphasis on female concerns regarding body image (Alleva et al., 2018; Guest et al., 2019). This result underscores the recognition of the challenges and unique experiences faced by women concerning multiple facets of body image and the need to address these issues specifically. It is worth noting that a significant portion of studies, approximately 28.6%, included samples with both women and men, emphasizing the importance of understanding body image relationships in both groups (Bassett-Gunter et al., 2017; Dryden & Anderson, 2019; Gattario & Frisén, 2019).

However, it is important to emphasize that the considerations require a critical analysis of the inherent limitations of studies on body image, particularly in terms of the omission of considerations of inclusion and intersectionality. Only one study focused on understanding how participants feel, identify, and present themselves, both to themselves and to those around them, using self-declared gender identity (for example, Bolter et al., 2023). In a recent study, Landor et al. (2024) advanced the structuring and conceptualization of the sociostructural-intersectional model of body image, with the aim of more fully understanding the body image experiences of those with racialized and marginalized bodies. Furthermore, they challenged and sought to subvert white supremacy for a better understanding of bodily inequities and the body image experiences of all individuals, in all bodies.

Historically, research and interventions related to body image have focused on the experiences of white, cisgender, heterosexual, and healthy women, many of whom are representative of university contexts in economically developed countries and Western cultures. This centralization has contributed to the perpetuation of notions of invulnerability among these groups and has established white norms and experiences as the benchmarks by which marginalized bodies are evaluated (Landor et al., 2024).

In addition to the aforementioned issues, the analysis of the studies included in this systematic review also revealed a clear predominance of quantitative methods in research on interventions focusing on the body image of university students. This preference indicates an emphasis on collecting numerical data and statistical analysis to investigate various aspects related to body image (e.g., Alves et al., 2017; Benitez et al., 2019). The quantitative approach allows for the objective measurement of phenomena and the establishment of relationships between variables, contributing to an understanding of the effects of interventions. It is noteworthy that two studies used a qualitative-quantitative method, and other two studies used a qualitative method. The qualitative-quantitative method allows for a deeper and richer understanding of the studied phenomena, combining the collection and analysis of quantitative data with the exploration of subjective and qualitative aspects. Harriger et al. (2023) suggested the use of mixed methods to achieve a comprehensive understanding of the effects of social media on body image, which, in our perspective, could be extended to other aspects related to body image, such as interventions.

The assessment instruments used in interventions cover a variety of body image constructs. The use of these instruments allows for a comprehensive evaluation of the effects of interventions in this area. However, it is essential to consider the limitations of assessment instruments, such as potential self-report biases, subjective interpretation, and cultural influences, for example. Careful selection of instruments, considering their psychometric properties, validity, and reliability, is crucial to ensure valid and reliable results. Future research can benefit from using more specific and sensitive instruments for body image constructs, as well as exploring new instruments that address emerging aspects of body image, such as the impacts of social media.

Finally, the inherent limitations of the methodologies employed in studies on interventions focused on the body image of university students have significant implications for interpreting the results. The predominance of quantitative methods, while allowing for the objective measurement of phenomena and the establishment of relationships between variables, may fail to capture the complexity and subjectivity of individual experiences related to body image. This quantitative focus tends to overlook qualitative aspects that are essential for a deeper understanding of the psychosocial dynamics involved.

Additionally, the use of predominantly female samples from Anglo-Saxon countries limits the generalization of findings to other populations, especially those from diverse cultural and social contexts. The lack of considerations regarding inclusion and intersectionality in the analyzed studies also points to a partial view that does not encompass the full range of experiences of marginalized groups. This centralization of experiences of white, cisgender, heterosexual women can perpetuate norms and standards that are not representative of the experiences of all individuals. Therefore, the methodologies adopted, by not considering these critical variables, may result in biased and incomplete interpretations, underscoring the need for more inclusive and diverse methodological approaches in future research.

### Intervention approach

In the realm of intervention approach in the investigated studies, a diversity of approaches aiming to explore the complex issues of body image of university students has emerged. In this context, a notable prevalence was observed in the adoption of the cognitive-behavioral approach, which was present in seven distinct studies (Aboody et al., 2020; Becker et al., 2013; Cerea et al., 2021; Dondzilo et al., 2020; Glashouwer et al., 2020; Ong & Sündermann, 2022; Yager & O’Dea, 2010). This approach aims to modify maladaptive cognitive and behavioral distortions related to body image, seeking to promote a healthier and more realistic view of one’s body, as emphasized and suggested in some recent studies (Betz et al., 2019; Halliwell et al., 2015).

Anchored in the fundamental premise that dysfunctional thoughts and behaviors inherent to body perception can be transformed, the cognitive-behavioral approach focuses on cognitive restructuring (Alleva et al., 2015). Although positive results have been documented in various studies employing this approach, it is essential to note that the effectiveness of interventions did not manifest uniformly. This variability suggests that the effectiveness of the cognitive-behavioral approach may be susceptible to variations due to the particularities inherent to the examined sample and the intensity of the implemented intervention, i.e., the number and duration of sessions.

In this regard, a more detailed analysis of contextual factors that can modulate the effects of this approach is necessary, emphasizing the importance of a personalized and adaptive approach. The study by Ong and Sündermann (2022) employed a cognitive-behavioral approach-based intervention through a mobile application to examine its effectiveness in improving the body image and self-compassion of university students. Despite promising initial results, it is necessary to consider that the attenuation of concerns about body image was not uniform, highlighting the influence of individual characteristics on the impact of the intervention.

Similarly, Cerea et al.’s (2021) research employed a mobile application based on cognitive-behavioral principles aiming to reduce body dissatisfaction and symptoms of body dysmorphic disorder in university students. The results indicated that the intervention led to reductions in body dissatisfaction and symptoms of body dysmorphic disorder; however, once again, the variability in results raises questions about the mediating factors of intervention efficacy.

Among the examined studies, some yielded results that did not reach satisfactory levels of effectiveness concerning the cognitive-behavioral intervention used. An example is the study conducted by Glashouwer et al. (2020), which employed a personalized avoidance approach training using participants’ body images. The goal was to test whether the training could improve body satisfaction. However, the results did not provide evidence that the avoidance-approach bias training was effective in altering negative body image. This illustrates that although the intervention approach in the aforementioned studies is the same, the results are not always as expected.

An approach that emerges as relevant in the context of the analyzed studies is media literacy, adopted in three distinct investigations (Khazir et al., 2015; Ong & Sündermann, 2022; Yager & O’Dea, 2010). This approach aims to enhance individual capacity to critically interpret messages and images conveyed in the media. It is noteworthy that the results obtained through this approach are promising.

The study conducted by Khazir et al. (2015) observed a significant reduction in favorable attitudes toward elective cosmetic surgery, indicating that media literacy contributed to the alteration of these perceptions. Additionally, the study by Yager and O’Dea (2010) explored the effects of a controlled intervention to promote positive body image. This study demonstrated the ability of media literacy to collaborate in reducing body dissatisfaction among university students. Both results not only reinforce the viability of media literacy, which emerges as an important approach among others, but also highlight the relevance of developing critical media analysis skills as an effective component in counteracting the influences it exerts.

Another approach that deserves attention within the panorama of the analyzed studies is mindfulness, which is present in three other research studies (Adams et al., 2013; Díaz-Ferrer et al., 2017; Toole & Craighead, 2016). These interventions are characterized by emphasizing mindfulness training, aiming to enhance the capacity to accept and be aware of the present moment. The investigation conducted by Toole and Craighead (2016) exemplifies such an approach by assessing the effects of a self-compassion meditation-based intervention. The results suggest that brief exposure to self-compassion principles could potentially improve aspects of self-compassion and reduce body discomfort.

Similarly, the study by Díaz-Ferrer et al. (2017) investigated the impact of mirror exposure training incorporating mindfulness elements. The results pointed to positive psychophysiological changes associated with mirror exposure, reinforcing the notion that mindfulness can contribute to modifying body image-related disorders. Nevertheless, it is crucial to note that the effectiveness of these mindfulness-based interventions may be sensitive to the regularity of practice and the level of participant engagement. The interpretation of these findings emphasizes the importance of considering both consistency in implementing mindfulness practice and the inclusion of strategies that promote participant adherence, thus expanding the potential of this approach to contribute to the promotion of positive body image.

Among the selected studies in this systematic review, other approaches also stood out for their contributions related to the body image of the individuals involved. The physical training approach, for example, was explored in studies such as Moore et al. (2011) and Henry et al. (2006). While Moore et al. (2011) adopted a resistance exercise program, Henry et al. (2006) investigated the impact of a weightlifting course at a university. Both studies highlight the relevance of physical activity as a potential intervention to develop integrated and full-body image and self-esteem, especially among women.

Additionally, the study conducted by Kosma et al. (2024) analyzed the effects of physical theater, integrating a university semester with two 90-min sessions per week, in a population of eight participants. Using semi-structured interviews, the study sought to examine the effects of this intervention on body schema, including aspects such as body posture, awareness, confidence, and expression. The results highlighted the importance of physical theater in improving students’ body posture, confidence, and expression, emphasizing the need for integrated and holistic movement programs to enhance body schema. These findings reinforce the importance of varied and structured physical activities for the development of a positive and healthy body image.

Two other approaches that deserve attention are creative psychotherapy and yoga. The former, examined by Ariel-Donges et al. (2019), demonstrated its potential to promote a positive body image; through yoga sessions, university women reported improvements in body concerns. The latter, employed in the study by Lee et al. (2019), using creative movement revealed improvements in body dissatisfaction and mindfulness among participating university students. Yoga emphasizes the importance of holistic practices that address the individual as an integrated organism, with no separation of body and mind, and psychotherapy illustrates how creative expression can be explored to promote a healthier relationship with one’s body.

In summary, the analysis of different intervention approaches focusing on the body image of university students reveals a multifaceted and promising scenario. The cognitive-behavioral approach stands out as a widely used strategy, but the variation in the effectiveness of the analyzed interventions indicates the need for a deeper understanding of contextual factors that can influence the results.

The media literacy approach emerges as an important means to confront media influences. The ability to critically interpret media messages related to the body is vital in an era dominated by social media, reinforcing the importance of developing these skills to counteract sociocultural pressures (Miller et al., 2019; Tamplin et al., 2018). Mindfulness-based interventions offer a unique perspective by focusing on acceptance and mindfulness. While promising results are observed, the regularity of practice and participant engagement emerge as crucial factors for consistent benefits. Physical training, creative psychotherapy, yoga, and physical theater present encouraging results, highlighting the importance of holistic approaches.

The diversity of approaches reveals the complexity of the relationship between interventions and their outcomes. It seems that there is no single approach that works for all contexts and individuals, emphasizing the need for a personalized approach adapted to the characteristics and needs of each target population, i.e., individual, contextual, and cultural particularities should be considered in interventions focusing on the body image of university students.

### Sessions, follow-up, and intervention and control groups

A detailed analysis of the studies in this systematic review reveals a notable heterogeneity regarding the number of sessions implemented in different interventions and the follow-up period. In the study conducted by Ong and Sündermann (2022), a mental health mobile app was used to provide training with cognitive-behavioral, psychoeducation, and media literacy approaches. The intervention spanned 9 days, with sessions of less than 5 min each day and a follow-up of 4 weeks. Preliminary results suggest that this self-guided intervention can contribute to body image and self-compassion among young adult university students.

Similarly, two other studies (e.g., Aboody et al., 2020; Cerea et al., 2021) also conducted studies using cognitive-behavioral training delivered through a mobile app. The intervention proposed by Cerea et al. (2021) lasted for 16 days, with 3-min sessions per day and a follow-up of 32 days. The goal was to evaluate the effectiveness of the intervention in reducing body dissatisfaction and symptoms of body dysmorphic disorder/eating disorders in female university students. The intervention conducted by Aboody et al. (2020) lasted for 14 days, with 4-min sessions per day and a 1-month follow-up. The aim was to assess the effects of the app in increasing resilience to body image triggers and reducing symptoms of body image disturbance. Both proved effective according to the proposed objectives.

On the other hand, Glashouwer et al. (2020) tested a personalized approach to avoidance training in university women over four sessions in 1 week. The results did not provide evidence that the avoidance-approach training was effective in improving participants’ negative body image, indicating a need for a greater number of sessions in this specific intervention. An intervention that caught attention was conducted by Toole and Craighead (2016), testing an Internet-based self-compassion training version over 1 week. Although the intervention was brief, the results indicated improvements in self-compassion and body discomfort among participants, suggesting that even short interventions can be effective in addressing the abovementioned issues.

In contrast, Khazir et al. (2015) conducted media literacy training over 4 weeks, one session per week lasting 40 to 60 min each. The results indicated a reduction in favorable attitudes toward cosmetic surgery, body dysmorphic disorder, and body dissatisfaction, along with an increase in self-esteem. This highlights and suggests that more extended engagement interventions can also be effective in achieving changes in participants’ body image.

In studies with longer interventions (e.g., Ariel-Donges et al., 2019; Henry et al., 2006; Kosma et al., 2024; Moore et al., 2011; Rato & Alves, 2020; Yager & O’Dea, 2010), only one study used a follow-up period. The study conducted by Yager and O’Dea (2010) examined the impact on body image, risk of eating disorders, and excessive exercise in 170 physical education teachers in training, using 6 months as the follow-up period. The researchers demonstrated the effectiveness of an intervention through a health education curriculum. The other studies, even without a follow-up period, presented advantages and positive aspects regarding the use of each intervention.

The relationship between the number of sessions and the results obtained, in our view, is quite complex. Studies with short intervention durations (e.g., Adams et al., 2013; Becker et al., 2013; Díaz-Ferrer et al., 2017; Dondzilo et al., 2020; Lee et al., 2019; Russell-Mayhew et al., 2012; Wilver et al., 2020) ranging from one to eight sessions also show effective results in their interventions. Only one study, conducted by Glashouwer et al. (2019), which had two sessions of 30 min each, indicated that the intervention format is not ready to be used as a strategy to improve participants’ body image.

In general, the discussion about the number of sessions and the follow-up period emphasizes the need for individualized and adapted approaches to fully develop the body image of university students. Interventions vary in duration and intensity, and it is crucial to consider factors such as specific objectives and the body image constructs addressed when designing these interventions. Additionally, the evaluation of long-term results and the maintenance of benefits over time are also important aspects to be explored in future research.

Considering the variation in the number of sessions and follow-up periods among the studies, it is possible to infer that there is no single ideal approach for all people and objectives. Shorter interventions may have specific positive effects, such as modifying attention bias patterns (e.g., Dondzilo et al., 2020), while longer interventions, such as yoga (Ariel-Donges et al., 2019) and physical training programs (Henry et al., 2006; Moore et al., 2011), may lead to more lasting benefits concerning body image. It is worth noting that the follow-up period varies widely among the studies analyzed in this systematic review, and future studies could explore the long-term maintenance of benefits from some interventions.

Ultimately, experimental and control groups play a fundamental role in analyzing interventions by providing essential information to determine if the proposed strategies are effective and relevant to the study. Some studies sought to understand the effects of interventions through carefully crafted comparisons between groups that received active treatment and those that did not. A similar number of participants were observed in the experimental and control groups of all studies that used this strategy, allowing for results evaluation considering the influences of external and natural factors. It is essential to note that the inclusion of experimental and control groups is relevant according to each research’s objectives. For example, the study conducted by Rato and Alves (2020) did not use group separation as its goal was the development and application of a Somatic Movement program to a group of undergraduate dance students, focusing on movement perception, and the study conducted by Kosma et al. (2024) also did not separate groups as the objective of the intervention was to examine the effects of a physical theater class on the participants’ body schema, evaluating body posture, awareness, confidence, and expression through qualitative aspects.

### Objectives and conclusion

Based on the analysis of the included studies, it becomes apparent that the conclusions were not always directly proportional to the initially defined objectives. While many studies yielded positive results regarding the effectiveness of interventions in the comprehensive development of body image among university students, some research also reported less conclusive outcomes.

As mentioned previously, the diversity of interventions is a striking feature of the studies included in this systematic review. Interventions range from mobile applications and computer-based training to physical exercise programs, yoga, and creative movement. This variety may reflect the complexity of the focus on body image and the understanding that there is no one-size-fits-all intervention.

Three studies stand out for aiming to explore the effectiveness of mobile applications based on cognitive-behavioral approaches. One focused on improving body image and self-compassion in university students (e.g., Ong & Sündermann, 2022), the second on reducing body dissatisfaction and symptoms of body dysmorphic disorder in female university students (e.g., Cerea et al., 2021), and another on increasing resilience to body image triggers and reducing symptoms of body image disturbance (e.g., Aboody et al., 2020). These studies concluded that these low-intensity interventions can be effective, suggesting they might be a viable option to reach many individuals.

On the other hand, the study by Glashouwer et al. (2020), which tested the effectiveness of thinness-related approach and avoidance training in improving body satisfaction, concluded that this intervention did not provide evidence of being effective in improving participants’ negative body image. Additionally, the study by Glashouwer et al. (2019), which investigated the effectiveness of an evaluative conditioning procedure on body satisfaction, concluded that the procedure was not ready to be used as an intervention to improve body satisfaction. Both findings underscore the importance of carefully examining the specific strategies used in each intervention and recognizing that not all interventions produce the same results.

Furthermore, two studies (e.g., Becker et al., 2013; Wilver et al., 2020) examined the relationship between appearance-related safety behaviors and body image. These studies suggest that addressing these behaviors directly may be crucial to interrupting the cycle of body dissatisfaction and concerns about physical appearance among participants.

Moreover, it is interesting to note that some interventions that focused on physical training (e.g., Henry et al., 2006; Russell-Mayhew et al., 2012), yoga (e.g., Ariel-Donges et al., 2019), and mindfulness (e.g., Adams et al., 2013; Díaz-Ferrer et al., 2017; Toole & Craighead, 2016) are examples of studies in which the objectives sought to promote a healthier and more compassionate relationship between participants and their bodies and succeeded in achieving their goals.

Studies in which the intervention resource consisted of university courses demonstrated the importance and effectiveness of pedagogical and physical approaches in improving body image among university students. The study by Kosma et al. (2024) examined the effects of a semester-long physical theater class, concluding that participants emphasized the importance of physical theater in improving body posture, confidence, and expression, highlighting the need for integrated movement programs. Bolter et al. (2023) evaluated the impact of a brief pedagogical intervention to address weight stigma among first-year university students, highlighting the importance of these interventions. Although the quantitative results were not significant, the qualitative data suggested that brief interventions integrated into existing courses could engage students.

Another example is the study by Rato and Alves (2020), which focused on a Somatic Movement program integrated into the dance bachelor’s curriculum, revealing that the conscious and combined use of multiple methods provided significant advantages during the intervention. Moore et al. (2011) investigated a 12-week resistance training program, demonstrating significant improvements in self-perception constructs at all levels of the Exercise and Self-Esteem Model. Finally, Yager and O’Dea (2010) examined the impact of interventions to promote healthy body image among physical education and health education trainee teachers, showing the feasibility of promoting body image, reducing body dissatisfaction, and excessive exercise through a health education curriculum.

In terms of limitations, many studies have relatively small samples and, therefore, may have limited statistical power to detect significant effects (e.g., Cerea et al., 2021; Díaz-Ferrer et al., 2017). Additionally, as the studies focus on a specific population, the generalization of results is limited. Therefore, when evaluating the studies and their conclusions, it is evident that interventions vary in terms of effectiveness, but overall, the results point to the relevance of facilitating the comprehensive development of body image among university students. Even though not all studies fully achieved their objectives, the diverse interventions explored indicate a positive step toward better understanding and addressing this issue. Furthermore, it highlights an ongoing need for research to identify the most effective and personalized ways to meet the individual needs of participants.

### Intervention resources

In a chronological view, the systematic review of studies revealed a significant increase in the use of digital technology as a resource for interventions focused on the body image of university students. In the last 5 years, out of the nine interventions analyzed, five utilized digital technology, whether in the form of a mobile application or computer. It is noteworthy that in the years 2023 and 2024, the interventions were integrated into the academic semester in the format of classes.

In this context, three studies used mobile applications as intervention resources (e.g., Aboody et al., 2020; Cerea et al., 2021; Ong & Sündermann, 2022). Ong and Sündermann (2022) developed an intervention using a mental health application called Intellect, offering content based on cognitive-behavioral approaches, psychoeducation, and media literacy. The studies conducted by Cerea et al. (2021) and Aboody et al. (2020) similarly used a mobile application, but the approach in both was purely cognitive-behavioral.

Another digital technological resource used is the computer. Three studies utilized this resource to perform tasks proposed by the interventions (e.g., Dondzilo et al., 2020; Glashouwer et al., 2019, 2020). The study conducted by Glashouwer et al. (2020) is a personalized approach-avoidance training with images of the bodies of the participants. The research by Dondzilo et al. (2020) proposed attention bias modification based on a touch screen, while the study conducted by Glashouwer et al. (2019) is a computer-based evaluative conditioning procedure with the use of positive social feedback. It is worth noting that of these three interventions, only the one conducted by Dondzilo et al. (2020) obtained effective results.

The interventions conducted by Kosma et al. (2024) and by Bolter et al. (2023), both from the United States, illustrate a notable trend in integrating interventions into the academic semester as part of university classes. Kosma et al. (2024) implemented a physical theater intervention with a small group of eight participants, incorporating the intervention into the semester’s coursework. Similarly, Bolter et al. (2023) conducted a brief weight bias pedagogical intervention with a diverse group of 81 participants, including women, men, transgender men, transgender women, and gender nonconforming individuals. This intervention was also embedded within the academic semester. These examples suggest a growing trend toward utilizing class-integrated interventions during the university semester, potentially enhancing the accessibility and impact of these programs on students’ body image.

These examples indicate that the use of digital technology as an intervention resource is seemingly a growing trend, at least in the realm of interventions focused on the body image of university students. The use of digital technology offers advantages such as greater accessibility and flexibility of time. However, it is essential to consider the limitations and challenges associated with this resource in this context, such as the lack of direct professional supervision, the need for adequate access to the technology in question, and the potential for emotional disconnection. Therefore, it is crucial that interventions using digital technology as a resource are developed with a solid theoretical foundation, scientific rigor, and careful evaluation of their effectiveness.

The examples indicate that integrating interventions into university semester classes and using digital technology as an intervention resource seem to be a growing trend, at least in the context of interventions focused on the body image of university students. The use of digital technology offers advantages such as greater accessibility and flexibility of time. However, it is essential to consider the limitations and challenges associated with this resource in this context, such as the lack of direct professional supervision, the need for adequate access to the technology in question, and the potential for emotional disconnection. Incorporating interventions into the class format provides a more systematic structure and can facilitate continuous student participation. However, it also requires academic institutions to adapt their curricula and ensure that the professionals involved are adequately prepared to monitor and support students during the interventions. This evolution highlights the importance of adapting interventions to social and cultural changes in the pursuit of their effectiveness.

Despite the relevant findings presented in this review, we need to consider some inherent limitations in the described research, including the following: (1) the sample composed of university students may limit the generalization of results to the young adult population (e.g., Ong & Sündermann, 2022); (2) small sample size (e.g., Cerea et al., 2021); (3) restricted sample of female students; (4) participant engagement and interpretability of results (e.g., Glashouwer et al., 2019); and (5) the need for long-term follow-up measures (e.g., Adams et al., 2013). In summary, the heterogeneity of limitations among the studies underscores the importance of interpreting the results with caution and the need for future research that is increasingly robust in methodological aspects.

This systematic review highlights the multifaceted nature of interventions focused on the body image of university students, revealing a variety of approaches ranging from cognitive-behavioral methods to mindfulness and physical training. The use of digital technology plays a prominent role, emphasizing the trend toward accessible and wide-reaching solutions. These interventions are critical because they address body image issues that are increasingly prevalent among university students and have potential long-term psychological and social implications. The diversity in approaches underscores the complexity of body image problems and suggests that no single method is universally effective, pointing to the need for tailoring interventions to specific cultural, contextual, and individual needs.

Future research should continue to explore and refine these intervention strategies to establish more effective methodologies that are sensitive to the diverse needs of the university population. Studies should particularly focus on the long-term effectiveness and sustainability of outcomes, integrating longitudinal follow-ups to assess the enduring impact of interventions. There is also an urgent need to expand research demographics beyond predominantly female, Anglo-Saxon populations to include more diverse cultural and social contexts, thus enhancing the generalizability of the findings. Additionally, the integration of qualitative methods could provide deeper insights into the subjective experiences of participants, enriching our understanding of interventions focused on body image.

For policymakers, the findings suggest the importance of incorporating evidence-based interventions into university programs and curricula. Practitioners should consider adopting a variety of intervention approaches to meet the diverse needs of their student populations. Moreover, researchers are encouraged to develop and test interventions that are culturally and contextually adapted, ensuring that these initiatives are inclusive and representative of all segments of the student body. Finally, all stakeholders should consider the implications of digital technology in delivering these interventions, ensuring that these platforms are effective, accessible, and ethically designed to support the well-being of students.

## Conclusion

This systematic review aimed to analyze the scientific production related to interventions in the body image of young university students, adopting rigorous standards. Studies highlighted a significant predominance of research originating from the United States, emphasizing the lack of investigations in the Brazilian context, and showing the need for more research on the subject from this country.

The participants’ demographics mainly focused on female experiences, although both genders were incorporated. The preference for quantitative methods was evident, emphasizing the importance of objective assessment. However, the review also suggested the possibility of a qualitative-quantitative approach for a more comprehensive understanding of phenomena related to body image.

The cognitive-behavioral approach stood out as prevalent. Other approaches, such as media literacy and mindfulness, showed promising results. The detailed analysis of the studies revealed diversity in the characteristics of interventions, indicating the absence of a single approach to body image issues, with effectiveness influenced by various factors such as goals, target population, and intensity.

In conclusion, this review highlights the difficulty in generalizing results due to variability in intervention effectiveness. Some were effective, while others did not achieve their goals. Holistic approaches, such as physical training, yoga, and mindfulness, suggested the importance of a well-being-centered perspective.

Despite limitations, such as small samples, the results emphasized the relevance of interventions for the university population. The trajectory over the years showed an adaptation to social changes, with digital technology emerging as a prominent resource. However, this review highlights the ongoing need for critical evaluation of the effectiveness of these interventions and continuous improvement of approaches, aligning them with contemporary needs. Further, to expand current knowledge, fill identified gaps, and contribute to the development of more effective interventions in the realm of body image for university students, future studies need to diversify samples, considering not only gender but also other demographic characteristics such as ethnicity, sexual orientation, and gender identity. This would allow for a more comprehensive understanding of interventions in different groups. Additionally, studies should explore approaches that adopt qualitative-quantitative methodologies for a deeper and more holistic understanding of phenomena related to body image. This may include qualitative analyses to capture participants’ subjective perceptions not only about aspects inherent to body image but also about the experience undergone during the intervention.

In summary, this review contributes to the understanding of interventions in body image, highlighting the importance of comprehensive and adaptive approaches. Progress in this field is dependent on a careful balance between innovation and scientific grounding, aiming for a lasting positive impact on individuals’ lives.

## Data Availability

Not applicable for that section.

## Abbreviations

ABC: Appearance Behavior Checklist
AFAQ: Anti-Fat Attitude Questionnaire
BAAS: Beliefs About Appearance Scale
BAR: Body appearance rating
BAS: Body Appreciation Scale
BAS-2: Body Appreciation Scale-2
BDD-YBOCS-SR: Body Dysmorphic Disorder-Yale-Brown Obsessive–Compulsive Scale
BDI-2: Body Dissatisfaction Inventory-2
BIAQ: Body Image Avoidance Questionnaire
BIATQ: Body Image-Acceptance and Action Questionnaire
BICI: Body Image Coping Strategies Inventory
BIDQ: Body Image Disturbance Questionnaire
BIQ: Body Image Questionnaire
BISS: Body Image States Scale
BPS: Body Parts Satisfaction Scale
BSIQ: Body Shape Interrogation Questionnaire
BSQ: Body Shape Questionnaire
BSRI: Brief State Rumination Inventory
BSS: Body Satisfaction Scale
BULIT-R: Bulimia Test-Revised
CES-D: Center for Epidemiologic Studies Depression Scale
CSW: Cultural standards for women
DASS-21: Depression Anxiety Stress Scale-21
DEBQ: Dutch Eating Behaviors Questionnaire
DT: Drive for thinness
EAT: Eating Attitudes Test
EAT-26: Eating Attitudes Test-26
EDE-Q: Eating Disorder Examination Questionnaire
EDI: Eating Disorder Inventory
EDI-3: Eating Disorder Inventory-3
FPS: Fat Phobia Scale
FFMQ: Five Facet Mindfulness Questionnaire
FMI: Fat mass index
GSW: Global self-worth
IBSS-R: Interpersonal Body Comparison Scale-Revised
IQ: Interpretation questionnaire
MAEDS: Multidimensional assessment of eating disorders symptoms
MBSRQ-AS: Multidimensional Body-Self Relations Questionnaire
OBCS: Objectified Body Consciousness Scale
PANAS: Positive and Negative Affect Schedule
PASTAS: Physical Appearance State and Trait Anxiety Scale
PSAQ: Physical Self-Appreciation Questionnaire
PSPP: Physical Self-Perception Profile
QDC: Questionnaire on Dysmorphic Concerns
QSU: Questionnaire of Smoking Urges
RSES: Rosenberg Self-Esteem Scale
SATAQ: Sociocultural Attitudes Toward Appearance Questionnaire
SATAQ-4: Sociocultural Attitudes Toward Appearance Questionnaire-4
SCS: Sociocultural standards
SISE: Single-Item Self-Esteem Scale
SOC: Sense of coherence
SPIN: Social Phobia Inventory
SSES: Self-esteem scale
SSQ: Self-Stat Body Image Questionnaire
TMS: Thinness motivation scale
VAS: Visual analog scale
